# Functional expression of Tim-3 on blasts and clinical impact of its ligand galectin-9 in myelodysplastic syndromes

**DOI:** 10.18632/oncotarget.21492

**Published:** 2017-10-04

**Authors:** Toshio Asayama, Hideto Tamura, Mariko Ishibashi, Yasuko Kuribayashi-Hamada, Asaka Onodera-Kondo, Namiko Okuyama, Akiko Yamada, Masumi Shimizu, Keiichi Moriya, Hidemi Takahashi, Koiti Inokuchi

**Affiliations:** ^1^ Department of Hematology, Nippon Medical School, Tokyo, Japan; ^2^ Department of Microbiology and Immunology, Nippon Medical School, Tokyo, Japan

**Keywords:** myelodysplastic syndromes, Tim-3, galectin-9, acute leukemia, immune checkpoint molecule

## Abstract

T-cell immunoglobulin mucin-3 (Tim-3), an inhibitory immune checkpoint receptor, is highly expressed on acute myeloid leukemia cells and its ligand galectin-9 is reported to drive leukemic progression by binding with Tim-3. However, it remains unclear whether the Tim-3–galectin-9 pathway is associated with the pathophysiology of myelodysplastic syndromes (MDS). Thus, we investigated the expression and function of Tim-3 and the clinical impact of its ligand galectin-9 in MDS. Tim-3 expression levels on MDS blasts by CD45/side-scatter or CD34/CD45 gating were increased as MDS progressed to the advanced stage. Tim-3 expression in the MDS blasts was upregulated in the presence of the cell culture supernatant of human stromal cells or the MDS-related cytokine transforming growth factor-β1. The proliferation of Tim-3^+^ MDS blasts was inhibited by the blockade of anti-Tim-3 antibody. Furthermore, plasma levels of galectin-9 were elevated as MDS progressed to the advanced stage in 70 MDS/acute leukemia transformed from MDS patients and was a prognostic factor in 40 MDS patients. Our data demonstrated that the Tim-3-galectin-9 pathway is associated with the pathogenesis and disease progression of MDS. These findings provide new insight into potential immunotherapy targeting the galectin-9–Tim-3 pathway in MDS.

## INTRODUCTION

Myelodysplastic syndromes (MDS) are clonal hematologic stem cell disorders characterized by cytopenia and a high risk of progression to acute myeloid leukemia (AML). Acute leukemia transformed from MDS (AL-MDS) occurs in 10–40% of MDS patients, and those patients had poor prognosis with a median survival time of 4.7 months [1]. Thus, it is crucial to elucidate the mechanism of disease progression and leukemic transformation in MDS patients. In progression to advanced disease, clonal blasts gain aggressive behavior with more proliferative potential and fewer apoptotic cells compared with those in patients with low-grade disease. During disease progression, some gene mutations tend to be newly acquired [2]. The suppression of antitumor immunity such as PD-L1 upregulation on MDS blasts may be associated with AML progression [3, 4].

T-cell immunoglobulin mucin-3 (Tim-3), an immune checkpoint receptor, is expressed on CD4^+^, CD8^+^, and regulatory T cells and other immune cells such as monocytes, dendritic cells, and natural killer cells and inhibits T cell proliferation in antitumor immunity [5]. At least four ligands for Tim-3 have been identified, i.e., galectin-9, high mobility group box 1 (HMGB-1), Ceacam-1, and phosphatidylserine [5]. Galectin-9, a member of the lectin subfamily, is one of the tandem-repeat types of galectin and inhibits immune responses or activates inflammatory reactions [6, 7]. It contributes to tumorigenesis or tumor progression mediated by tumor cell transformation, cell-cycle regulation, angiogenesis, and cell adhesion [8]. Tim-3 was reported to form an autocrine loop with galectin-9 in AML cells [9]. However, it remains unclear whether these molecules are involved in the pathogenesis of MDS. In the current study, we investigated whether Tim-3 is associated with pathophysiological roles and whether the plasma galectin-9 concentration has clinical significance in MDS.

## RESULTS

### Tim-3 expression in MDS blasts

**Figure 1:**
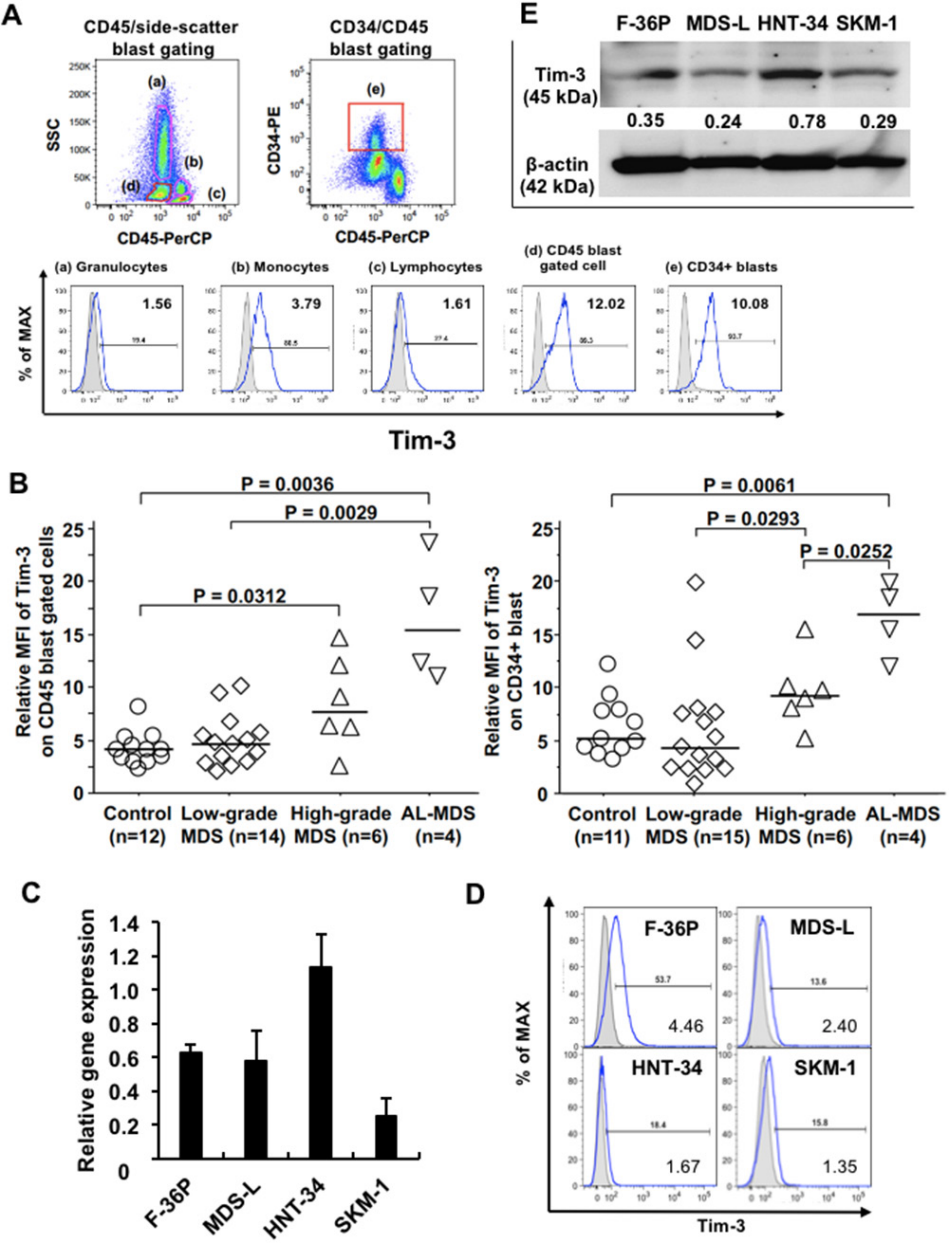
Tim-3 expression in MDS patients and MDS cell lines **(A)** Cell surface expression of Tim-3 in BM cells from an MDS patient was analyzed by FCM. Granulocytes (a), monocytes (b), lymphocytes (c), blasts (d), and CD34+ blasts (e) were gated using the CD45/side-scatter and CD34/CD45 gating methods. Solid line, staining with antibody to cell surface antigen; filled area, staining with isotype-matched control Ig. The numerical values in the lower right of each histogram are represented by relative MFI. **(B)** Comparison of cell surface Tim-3 expression on blasts among those from normal controls, patients with low-grade MDS (BM blasts <5%), high-grade MDS (BM blasts 5–19%), and AL-MDS. Tim-3 mRNA **(C)** and protein **(D)** expression in MDS cell lines was determined by qPCR and FCM, respectively. The data are mean ± SD. **(E)** Western blot analysis of Tim-3 and β-actin in MDS cell lines. Numbers under the Tim-3 band indicate the relative intensity of Tim-3 normalized to the signal intensity of β-actin. Tim-3, 45 kDa; β-actin, 42 kDa.

First, we investigated cell surface Tim-3 expression on granulocytes, lymphocytes, monocytes, and blasts in bone marrow (BM) cells obtained from patients with MDS and AL-MDS using flow cytometry (FCM). Obvious expression of Tim-3 was detected on blasts by both CD45/side-scatter and CD34/CD45 gating methods and on monocytes in BM cells (Figure 1A shows the results in an AL-MDS patient). Tim-3 expression levels on blasts from patients with MDS with ≥5% BM blasts and AL-MDS were significantly higher compared with those from hematologically normal controls (Figure 1B, Supplementary Figure 1A). Although the number of patients was limited, high Tim-3 expression was detected in AL-MDS patients. Next, we evaluated the mRNA and cell surface expression of Tim-3 in four MDS cell lines. *Tim-3* mRNA was expressed in all cell lines (Figure 1C), and the percentage of Tim-3^+^ cells was the highest in F-36P cells (Figure 1D), although Tim-3 protein expression in whole-cell lysis was detected in all cell lines by Western blotting (Figure 1E).

### Tim-3 induction in the BM microenvironment

**Figure 2:**
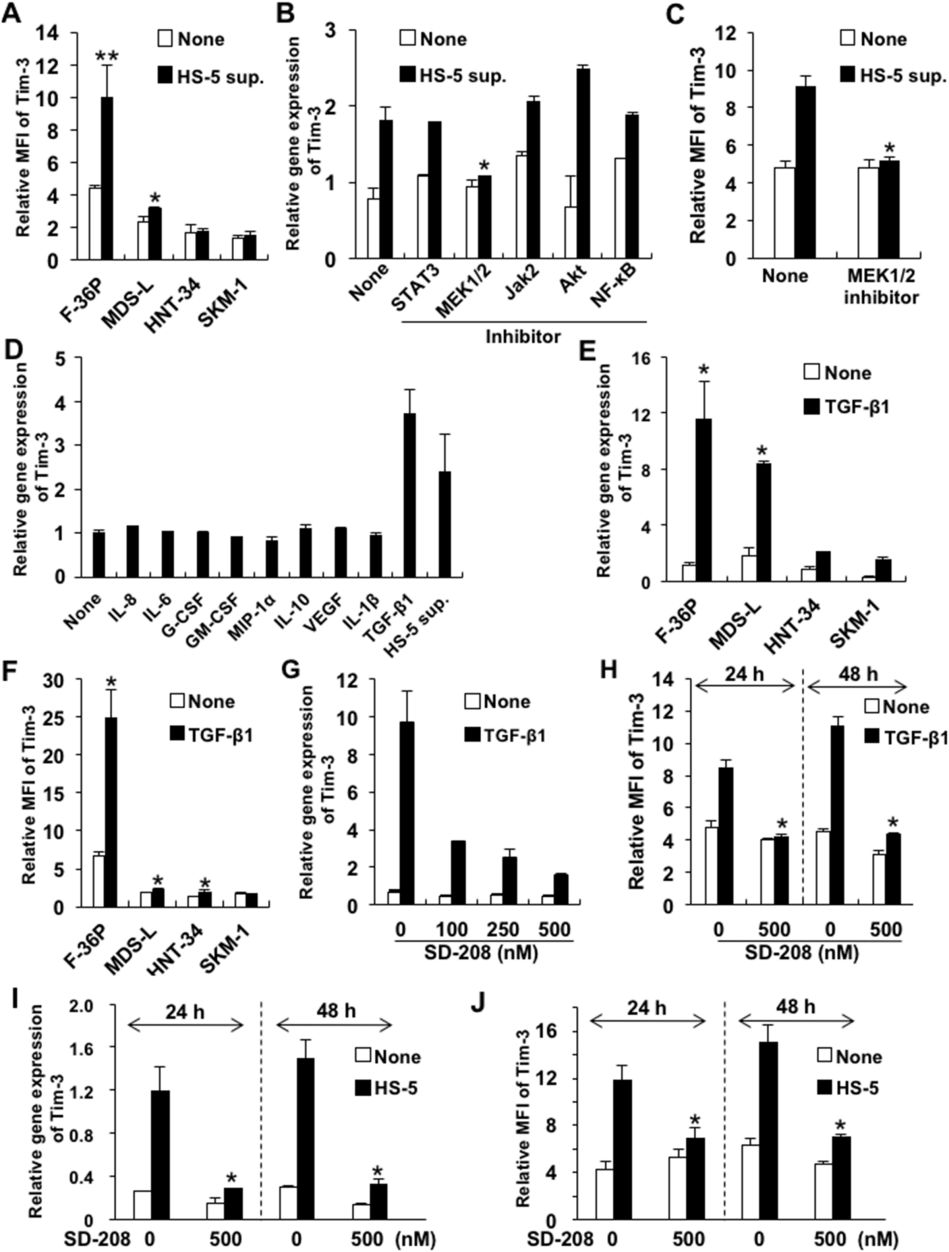
Upregulated Tim-3 expression in MDS cell lines **(A)** MDS cell lines were cultured with or without HS-5 sup. for 48 h. The cells were pretreated with signal transduction inhibitors of STAT3, MEK1/2, JAK2, Akt/PI3K, and NF-κB for 2 h and then cultured in complete medium containing HS-5 sup., after which Tim-3 mRNA expression **(B)** and protein were analyzed **(C)**. The concentrations of each inhibitor were 500 nM of STAT3 inhibitor V, 20 μM of U0126 (MEK1/2 inhibitor), 25 μM of AG490 (JAK2 inhibitor), 25 μM of LY294002 (PI3K/AKT signaling inhibitor), and 5 nM of PDTC (NF-κB inhibitor). **(D)** F-36P cells were cultured with the following cytokines for 2 days: 5 ng/ml of IL-8, 5 ng/ml of IL-6, 100 pg/ml of G-CSF, 10 ng/ml of GM-CSF, 10 ng/ml of MIP-1α, 10 ng/ml of IL-10, 10 ng/ml of VEGF, 10 ng/ml of IL-1β, and 2.5 ng/ml of TGF-β1. **(E, F)** MDS cell lines were cultured with 2.5 ng/ml of TGF-β1 for 48 h. **(G–J)** F-36P cells were pretreated with SD208, a selective inhibitor of TGF-βRI kinase, at optimal concentrations for 2 h, followed by incubation with 2.5 ng/ml of TGF-β1 (G, H) or HS-5 sup. (I, J) for 24–48 h. After incubation with HS-5 sup. or TGF-β1, the cell surface (A, C, F, H, J) and mRNA (B, D, E, G, I) expression of Tim-3 was analyzed by FCM and real-time qPCR, respectively. Data represent mean ± SD. *P<0.05, **P<0.005 compared with the results without HS-5 sup. or cytokines (A, E, F) and with the results without inhibitors (B, C, H, I, J).

To investigate whether Tim-3 expression on blasts could be induced by soluble factors in the BM microenvironment of MDS, we evaluated its expression on MDS cells cultured in complete medium containing culture supernatant of the human BM stromal cell line HS-5 (HS-5 sup.) or MDS-associated cytokines. Tim-3 expression was increased by HS-5 sup. in the F-36P and MDS-L cell lines (Figure 2A). To identify the intracellular signaling pathway of Tim-3 induction by HS-5 sup., we evaluated *Tim-3* mRNA expression in F-36P cells by HS-5 sup. in addition to various signal transduction inhibitors. The increases in *Tim-3* mRNA and cell surface expression induced by HS-5 sup. in F-36P cells were inhibited by the MEK 1/2 inhibitor U0126 (Figure 2B and 2C).

Next, we evaluated which major cytokines produced by HS-5, i.e., interleukin (IL)-6, IL-8, granulocyte-colony stimulating factor (G-CSF), granulocyte macrophage-CSF (GM-CSF), macrophage inflammatory protein (MIP)-1α, IL-1β [10], and MDS-associated cytokines, i.e., IL-10, vascular endothelial growth factor (VEGF), and transforming growth factor (TGF)-β1 [3, 11–13], could induce Tim-3 expression on F-36P cells. TGF-β1 alone enhanced *Tim-3* mRNA expression on F-36P cells (Figure 2D). *Tim-3* mRNA expression was clearly upregulated by TGF-β1 in all MDS cell lines (Figure 2E). However, while cell surface Tim-3 expression in F-36P cells was dramatically increased by TGF-β1, it was not in other MDS cell lines (Figure 2F). Similar to the results in F-36P cells, TGF-β1 or HS-5 sup. increased various levels of Tim-3 expression in MDS blasts obtained from 2 AL-MDS and 1 refractory anemia with excess blasts (RAEB) (RAEB-2) patients (Supplementary Figure 2). TGF-β1-induced upregulation of *Tim-3* mRNA in F-36P cells was inhibited dose dependently by SD208, a specific inhibitor of TGF-β receptor type 1 kinase (Figure 2G). Consistent with these results, Tim-3 expression on the cell surface was suppressed by SD208 at 24 and 48 h posttreatment (Figure 2H). Furthermore, Tim-3 mRNA and surface protein expression induced by HS-5 sup. was inhibited by SD-208 (Figure 2I and 2J). Those findings suggest that TGF-β1 is a major inducer of Tim-3 expression in F-36P cells.

### Gene expression and proliferative functions of Tim-3^+^ MDS blasts

**Figure 3:**
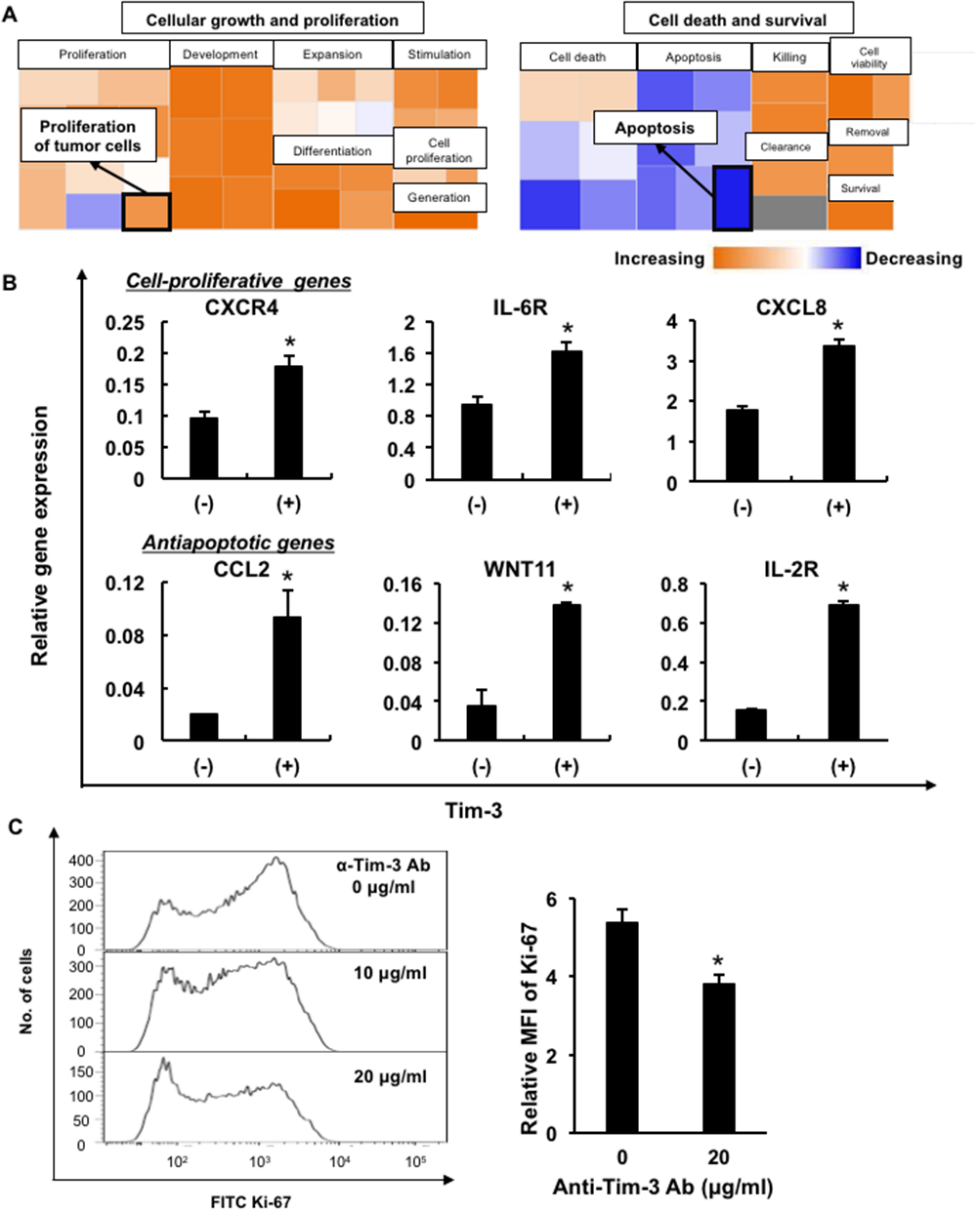
Upregulation of cell proliferation-related and antiapoptotic genes in Tim-3^+^ MDS cells **(A)** Comparison of mRNA expression of antiapoptotic genes and cell-proliferative genes in Tim-3^+^ and Tim-3^–^ F-36P cells. **(B)** The cell proliferation-related genes *CXCR4*, *IL-6R*, and *CXCL8*, and antiapoptotic genes *CCL2*, *WNT11*, and *IL-2R*, were validated using real-time PCR in Tim-3^+^ and Tim-3^–^ F-36P cells. **(C)** Ki-67 expression in F-36P cells pretreated with anti-Tim-3 blocking antibodies (α-Tim-3). After overnight incubation of treated cells, intracellular Ki-67 expression was evaluated using FCM. Representative data of more than three experiments are shown. *P<0.05 compared with the results of Tim-3^–^ F-36P cells (B) and with the results without the blocking antibody (C).

To elucidate the pathophysiological functions of Tim-3 in MDS, we used oligonucleotide microarrays to examine the differential gene expression between Tim-3^+^ and Tim-3^–^ cell fractions in F-36P cells (Supplementary Figure 3A). Five hundred seventy-two upregulated genes (>2-fold difference) and 304 downregulated genes (<0.5-fold difference) were identified in Tim-3^+^ F-36P cells. Ingenuity pathway analysis of those genes revealed upregulation associated with cell proliferation and antiapoptotic responses in Tim-3^+^ cells (Figure 3A and Supplementary Figure 3B). We confirmed upregulation of those genes in Tim-3^+^ cell fractions by real-time PCR, that is, the cell proliferation-related genes, *C-X-C chemokine receptor type 4 (CXCR4)*, *IL-6 receptor (IL-6R)*, and *C-X-C motif chemokine ligand 8 (CXCL8)*, and antiapoptotic genes, *C-C motif chemokine ligand 2 (CCL2)*, *Wnt family member 11 (WNT11)*, and *IL-2 receptor (IL-2R)* (Figure 3B).

To investigate whether the Tim-3 expression on blasts was involved in cell growth, intracellular Ki-67 expression was examined in F-36P cells after cultivation with blockade of the anti-Tim-3 antibody. Ki-67 expression was significantly decreased in F-36P cells after treatment with the anti-Tim-3 antibody (Figure 3C), suggesting that Tim-3 delivered proliferative signals in MDS blasts. Furthermore, Tim-3^+^ F-36P cells had more proliferative potential and resistance to the anti-MDS agent cytarabine in comparison with Tim-3^–^ cells (Supplementary Figure 4).

### Galectin-9 expression and its secretion from MDS blasts

**Figure 4:**
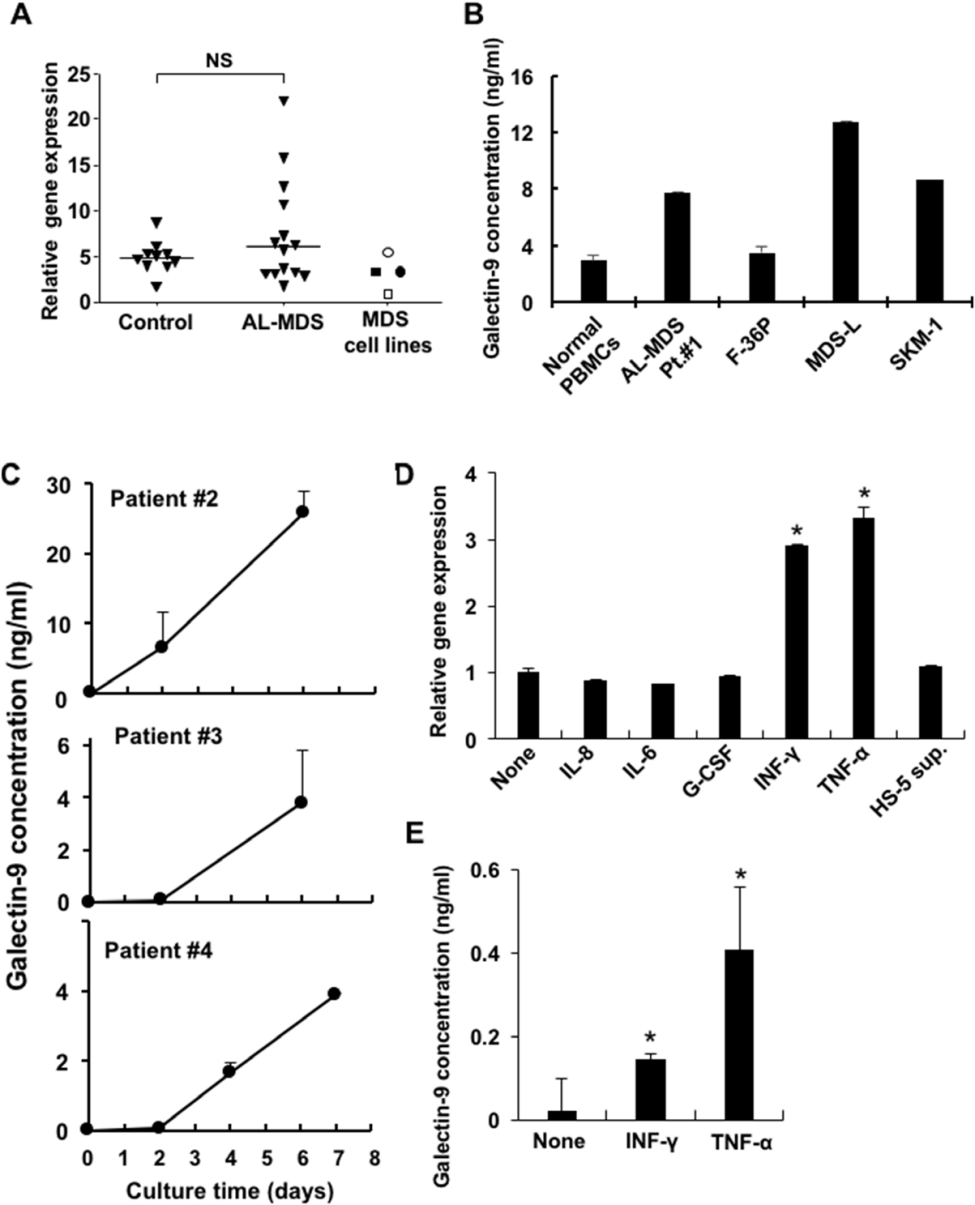
Expression and soluble form of galectin-9 in MDS patients and MDS cell lines **(A)** Galectin-9 mRNA in PBMCs obtained from healthy controls, AL-MDS patients (blasts >95%), and MDS cell lines was determined by real-time PCR. Open rectangle, F-36P; closed rectangle, MDS-L; open circle, HNT-34; closed circle, SKM-1. NS, not significant. **(B)** Soluble galectin-9 in cell culture supernatant of normal PBMCs (*n* = 3), patient blasts and MDS cell lines were determined by ELISA. 1 x 10^6^ cells/ml of MDS blasts or normal PBMCs and 2 x 10^5^ cells/ml of MDS cell lines were cultured in complete medium and the culture supernatants were harvested after 2 and 3 days of culture, respectively. **(C)** 1 x 10^6^ cells/ml of PBMCs obtained from AL-MDS patients were cultured and then the galectin-9 concentrations in culture supernatants acquired on days 2 and 6 (patients #2, #3) and days 2, 4, and 7 (patient #4) were analyzed using ELISA. **(D)** 2 x 10^5^ cells/ml of F-36P cells were co-cultured with each the following cytokines for 2 days: 5 ng/ml of IL-8, 5 ng/ml of IL-6, 100 pg/ml of G-CSF, 10 ng/ml of IFN-γ, and 500 U/ml of TNF-α and HS-5 sup. Then *galectin-9* mRNA expression was evaluated using real-time PCR. **(E)** Galectin-9 concentrations in the cell culture supernatant of F-36P cells treated with IFN-γ and TNF-α were quantified by ELISA. Data represent mean ± SD. *P<0.05 compared with the results without HS-5 sup. or cytokines (D, E).

Galectin-9 is well known to be an effective ligand of Tim-3 to deliver positive signals, and thus we evaluated *galectin-9* mRNA expression in peripheral blood mononuclear cells (PBMCs) obtained from healthy donors and AL-MDS patients (>95% blasts) and in MDS cell lines. There was no significant difference in *galectin-9* mRNA expression in PBMCs from controls and AL-MDS patients, but higher expression was detected in 4 of 14 AL-MDS patients (Figure 4A). The soluble form of galectin-9 was produced by MDS cell lines and in PBMCs obtained from an AL-MDS patient, although the levels were very low in the cell culture supernatants of PBMCs from normal controls (Figure 4B). Galectin-9 levels in the culture supernatants of PBMCs from 3 AL-MDS patients were markedly elevated in a time-dependent manner (Figure 4C). Next, to investigate whether galectin-9 secretion is enhanced in the MDS microenvironment, F-36P cells were cultured with interferon (IFN)-γ, tumor necrosis factor (TNF)-α, IL-8, IL-6, and G-CSF. After 2 days of culture, the expression of *galectin-9* mRNA was upregulated by IFN-γ or TNF-α (Figure 4D), and these cytokines also increased the production of galectin-9 (Figure 4E). However, those cytokines did not increase mRNA and protein expression of Tim-3 on MDS cell lines (data not shown).

### Plasma galectin-9 in MDS patients

**Figure 5:**
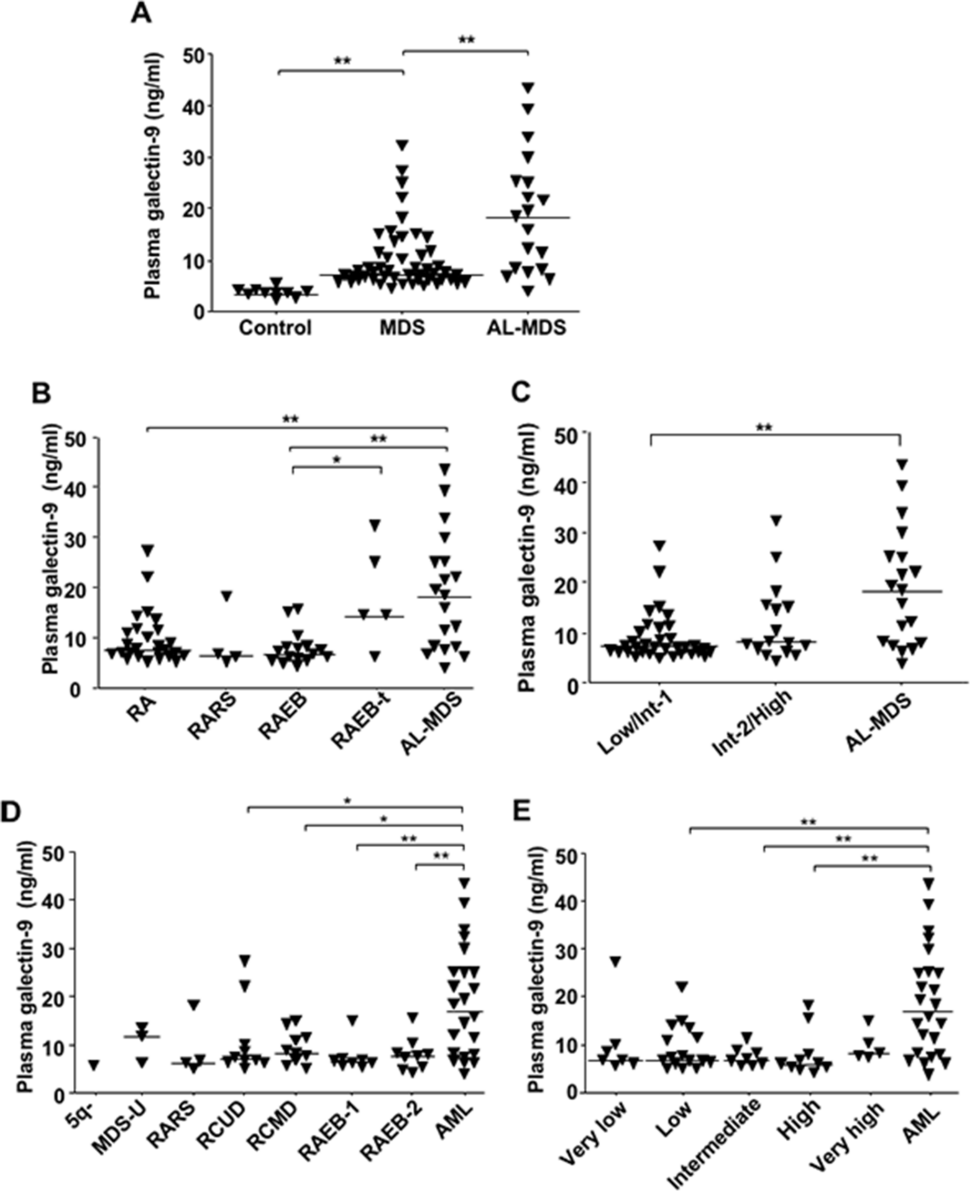
Soluble galectin-9 levels in MDS patients according to disease stage Plasma galectin-9 concentrations were measured using ELISA. **(A)** Galectin-9 concentrations in plasma from 10 healthy donors, 51 MDS patients, and 19 AL-MDS patients. **(B)** Comparison of plasma galectin-9 levels in MDS patients according to the FAB classification: RA (*n* = 26); RARS (*n* = 3); RAEB (*n* = 16); and RAEB in transformation (RAEB-t; *n* = 5). **(C)** Comparison of plasma galectin-9 levels in MDS patients according to the IPSS: Low/Int-1 (*n* = 33) and Int-2/High (*n* = 17). **(D)** Comparison of plasma galectin-9 level in MDS patients according to the WHO classification: 5q- (*n* = 1); MDS-U (*n* = 3); RARS (*n* = 3); refractory cytopenia with unilineage dysplasia (RCUD; *n* = 10); refractory cytopenia with multilineage dysplasia (RCMD; *n* = 11); RAEB-1 (*n* = 8); RAEB-2 (*n* = 9); and AML (*n* = 23). **(E)** Comparison of plasma galectin-9 levels in MDS patients according to the WPSS: very low (*n* = 7); low (*n* = 17); intermediate (*n* = 8); high (*n* = 9); and very high (*n* = 5). Data represent mean ± SD. *P < 0.05, **P < 0.01. Int-1, intermediate-1; Int-2, intermediate-2.

We evaluated concentrations of plasma galectin-9 in MDS and AL-MDS patients (Table 1 shows patient characteristics). The concentrations were significantly higher in 70 patients with MDS and AL-MDS than in healthy controls (Figure 5A). On the other hand, there was no significant difference in plasma levels of another ligand, HMGB-1, between healthy controls and patients with MDS and AL-MDS (Supplementary Figure 5). Plasma galectin-9 levels in AL-MDS patients were significantly elevated in comparison with those in patients with refractory anemia (RA) and RAEB (*P* = 0.0051 and *P* = 0.0013, respectively; Figure 5B). According to the International Prognostic Scoring System (IPSS) classification [14], plasma galectin-9 levels in AL-MDS patients were markedly higher than in low-risk MDS (IPSS low/intermediate-1) patients (*P* = 0.0006), but there was no significant difference in the level between low-risk and high-risk MDS (IPSS intermediate-2/high) patients (Figure 5C). There were no significant differences in plasma galectin-9 levels in MDS subtypes according to the World Health Organization (WHO) (Figure 5D) and WHO classification-based Prognostic Scoring System (WPSS) classifications (Figure 5E) [15]. However, the plasma galectin-9 level was significantly higher in patients with AML than in MDS patients.

**Table 1:** Patient characteristics

Median age (range)	70 (30–90)
Gender	
Female	28
Male	42
Diagnosis	
FAB classification	
RA	26
RARS	4
RAEB	16
RAEB-t	5
AL-MDS	19
WHO classification	
RCUD	10
RCMD	11
RARS	4
5q-	1
MDS-U	3
RAEB-1	8
RAEB-2	9
AML	24

FAB (French-American-British) classification: RA, refractory anemia; RARS, RA with ringed sideroblasts; RAEB, RA with excess blasts; RAEB-t, RAEB in transformation; AL-MDS, acute leukemia transformed from myelodysplastic syndromes (MDS). WHO (World Health Organization) classification: RCUD, refractory cytopenia of unilineage dysplasia; RCMD, refractory cytopenia of multilineage dysplasia; RARS, RA with ring sideroblasts; MDS-U, MDS, unclassified.

### Clinical impact of galectin-9 in MDS patients

**Figure 6:**
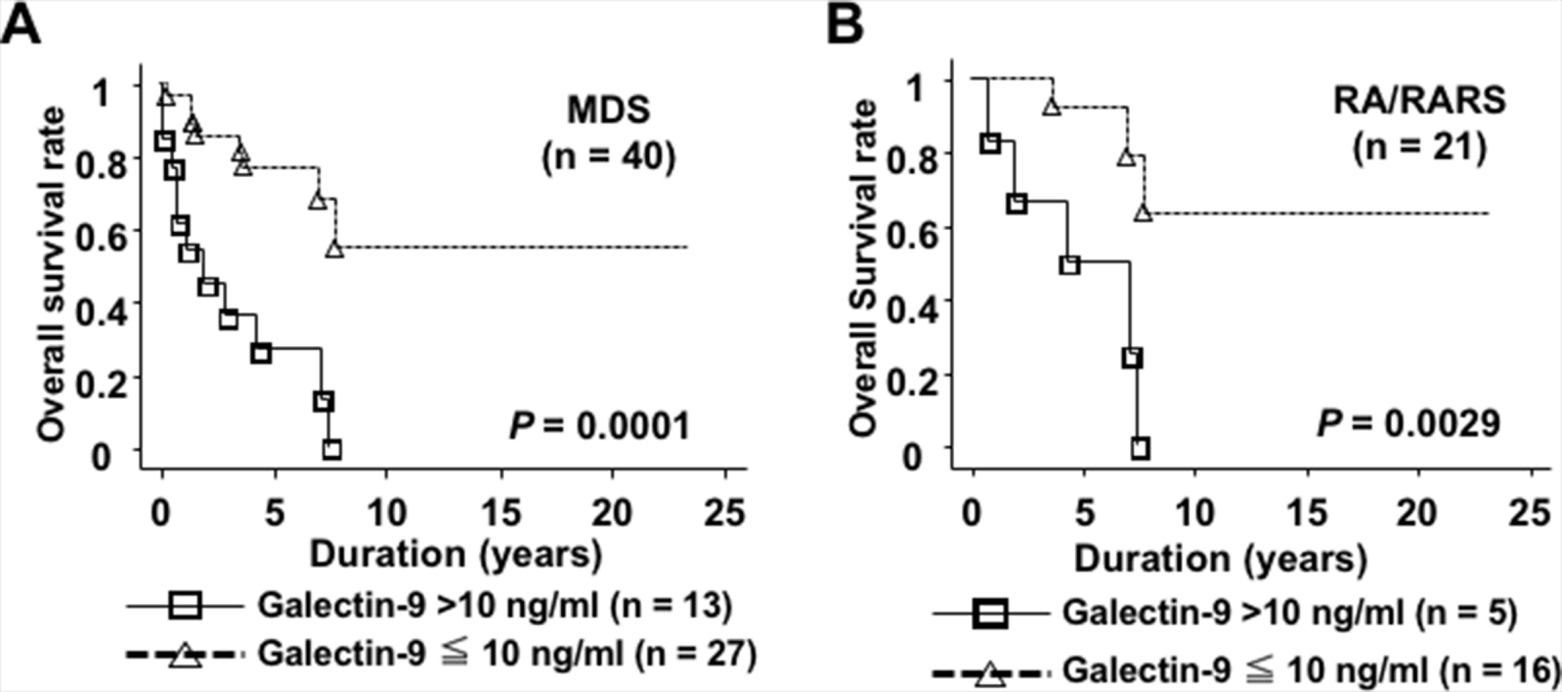
Plasma galectin-9 levels were associated with shorter OS times in MDS patients **(A)** The high galectin-9 group of MDS patients (>10 ng/ml, solid line) had shorter OS compared with the low group (≤10 ng/ml, dotted line). **(B)** In MDS patients with RA/RARS, the high galectin-9 group had shorter OS in comparison with the low group.

To assess the clinical impact of plasma galectin-9, we examined the differences in clinical characteristics between 2 groups according to its levels: high (>10 ng/ml, *n* = 13) and low (≤10 ng/ml, *n* = 27), because the galectin-9 levels were less than 10 ng/ml in healthy controls, similar to the manufacturer’s report. MDS patients in the high group tended to have fewer platelets and higher IPSS scores than those in the low group (Table 2). Overall survival (OS) time in patients in the high group was significantly shorter than in those in the low group (*P* = 0.0001, Figure 6A). In RA/RA with ringed sideroblasts (RARS) patients alone, OS rates in the high group were significantly lower than those in the low group (*P* = 0.0029, Figure 6B). The plasma galectin-9 level was an independent prognostic factor as well as the IPSS classification in both univariate and multivariate analyses (Table 3). These results suggest that high plasma galectin-9 levels could be associated with poor prognosis in MDS even in lower disease stages.

**Table 2:** Differences in clinical characteristics between the high and low plasma galectin-9 MDS patient groups

	>10 ng/ml	≤10 ng/ml	*P* value
Age (years) (range)	69 (36–80)	66 (30–83)	0.5442
Gender (M/F)	9/4	17/10	0.6971
Hb	7.78 ± 1.66	9.15 ± 2.62	0.2039
Plt (×10^4^/μl)	8.62 ± 8.84	14.4 ± 11.19	0.0782
WBC (×1000/μl)	10.5 ± 17.4	3.06 ± 1.65	0.3943
BM blasts	3.12 ± 6.13	0.59 ± 2.79	0.1408
IPSS			
High	6	3	
Intermediate-2	3	13	
Intermediate-1	2	4	0.0857
Low	2	7	

Hb, hemoglobin; Plt, platelets; WBC, white blood cell count; BM, bone marrow; IPSS, International Prognostic Scoring System.

**Table 3:** Results of univariate and multivariate analyses

Variable	Univariate analysis		Multivariate analysis	
	HR (95%CI)	*P* value	HR (95%CI)	*P* value
Plasma galectin-9 level(>10 ng/ml vs <10 ng/ml)	5.441	0.0005	4.939	0.0017
	(2.087–14.182)		(1.823–13.379)	
IPSS risk(Low/Int-1 vs Int-2/High)	6.789	0.0007	6.318	0.0020
	(2.253–20.460)		(1.965–20.314)	

HR, hazard ratio; IPSS, International Prognostic Scoring System.

## DISCUSSION

Tim-3 molecules were expressed on blasts from MDS patients with advanced-stage disease. Its expression was induced by soluble factors secreted by human stromal cells or TGF-β1 and associated with the proliferation of MDS cells. Furthermore, galectin-9, a ligand for Tim-3, was linked with shorter survival times in MDS patients. Our data demonstrated that Tim-3 on MDS blasts could be induced by the BM microenvironment and its ligand galectin-9 production may be induced in MDS patients with advanced-stage disease. This in turn suggests that the Tim-3–galectin-9 axis may lead to disease progression and leukemic transformation.

Tim-3, also known as an immune checkpoint molecule, is expressed on exhausted T cells and can deliver negative signals to T cells in patients with AML and MDS [16, 17]. Thus, in MDS patients, galectin-9 may induce T-cell apoptosis through Tim-3, which could be associated with tumor progression with immune escape. It was reported that Tim-3 is differentially localized in various types of cells. In primary AML cells, Tim-3 molecules were distributed largely on the surface. However, in healthy leukocytes, Tim-3 protein was mainly expressed intracellularly [18]. In MDS cells, Tim-3 localization differed in each cell line. HNT-34 cells expressed higher levels of mRNA and protein in whole cells compared with other MDS cell lines, with negligible levels of surface expression (Figure 1C–1E). In MDS blasts from an AL-MDS patient, intracellular Tim-3 expression levels were higher than surface expression levels (Supplementary Figure 1B). Although it remains unclear how the surface expression of Tim-3 is modulated [18], the distinct location of Tim-3 in MDS blasts may be caused by heterogeneous characteristics, such as cytokine production and signal transduction, in each cell line and patient.

The mechanism inducing the expression of Tim-3 and galectin-9 in MDS blasts definitely differs with the inductive cytokine. Galectin-9 was induced by proinflammatory IFN-γ and TNF-α, which were significantly increased in MDS patients [11]. On the other hand, Tim-3 expression on MDS blasts could be induced by TGF-β1, which has direct myelosuppressive effects and has been implicated in the autocrine production of IFN-γ and TNF-α in MDS [19]. Our results demonstrated that AL-MDS patients had higher expression levels of Tim-3 on blasts. In patients with advanced disease such as high-grade MDS and AL-MDS, the BM microenvironment may change with the upregulation of cytokines including TGF-β1, resulting in high Tim-3 expression. It was reported that Tim-3 is required for galectin-9 secretion, as it acts as a trafficker for galectin-9, in AML cells [18]. Tim-3 expression may be associated with galectin-9 production from MDS blasts, similar to AML cells.

Tim-3 is expressed on terminally differentiated Th1 cells and associated with T cell dysregulation by inducing apoptosis via ligation with galectin-9 [20]. In the BM microenvironment, Tim-3 in both CD4^+^ and CD8^+^ T cells was increased in AML patients [16]. Kikushige et al. reported that Tim-3 was expressed in the leukemic stem cell fraction (Lin^–^CD34^+^CD38^–^) and common progenitor fraction (Lin^–^CD34^+^CD38^+^) in AML patients, except for those with acute promyelocytic leukemia, while Tim-3 was undetectable on normal hematopoietic stem cells [21]. In MDS, Tim-3 expression levels on hematopoietic stem cells were higher than in normal controls [22]. In a limited number of our cases, AL-MDS patients had higher expression levels of Tim-3 on blast cells. Tim-3 expression may therefore increase as blasts become more proliferative, such as in AL-MDS. It was reported that galectin-9 secreted from leukemic cells ligated with Tim-3 and induced activation of the nuclear factor (NF)-κB and β-catenin signaling pathways in human AML cells, indicating that this pathway could be critical in the self-renewal of leukemic stem cells [9]. Darwish et al. demonstrated that blockade of Tim-3 inhibited the cell growth of AML cell lines [23]. As in AML cells, the blockade of Tim-3 inhibited the proliferation and cell growth of MDS cells (Figure 3C), suggesting that Tim-3 may contribute to tumor progression in MDS. However, it remains unclear whether the Tim-3–galectin-3 pathway could deliver NF-κB and β-catenin signaling.

We evaluated two major ligands for Tim-3, galectin-9 and HMGB-1, in MDS patients because our preliminary studies showed negligible expression of other ligands of Tim-3, i.e., Ceacam-1 and phosphatidylserine, on MDS cell lines (data not shown). Galectin-9 expression is widely distributed in several tissues in both physiological and cancerous states. In almost all solid cancers, galectin-9 expression was found to be decreased in cancerous tissues, except for oral and pancreatic cancer and melanoma [24–26]. In hematological malignancies, galectin-9 was implicated in the suppression of antitumor immunity by T cell exhaustion via the galectin-9/Tim-3 axis in murine AML models [27]. Our data demonstrated that plasma galectin-9 levels were associated with the disease progression of MDS. This may occur due to both induction of blast proliferation and suppression of antitumor immunity by T cell exhaustion via the galectin-9/Tim-3 axis in MDS. The ligand HMGB-1 may inhibit antitumor immunity through interactions with Tim-3 on tumor-infiltrating dendritic cells [28]. However, we found no difference in plasma HMGB-1 levels between patients with MDS/AL-MDS and healthy controls, suggesting that galectin-9 could be a critical ligand for Tim-3 on blasts in MDS.

Cell proliferation and antiapoptotic activity are key functions in disease progression and leukemic transformation in MDS. In Tim-3^+^ MDS cells, the cell proliferation-related genes *CXCR4*, *IL-6R*, and *CXCL8* and antiapoptotic genes *CCL2*, *WNT11*, and *IL-2R* were upregulated. The IL-6Rα chain, CXCR4, and CXCL8 could contribute to the proliferation of AML blasts, because inhibition of those molecules decreases the proliferative potential of AML blasts [29–31]. This supports the hypothesis that *CXCR4*, *IL-6R*, and *CXCL8* contribute to the cell proliferation of Tim-3^+^ blasts. *WNT11* mRNA overexpression was associated with poor prognosis in a subset of patients with AML [32], and thus *WNT11* may have an antiapoptotic function in Tim-3^+^ blasts in prostate and breast cancer [33, 34]. Plasma CCL2 upregulation in the peripheral blood (PB) and BM was reported in AML patients [35, 36], and CCL2 expression levels in the tumor microenvironment were elevated in MDS as well as AML [37]. CCL2 may decrease the apoptosis of Tim-3^+^ blasts as in neurons and astrocytes [38]. The cell surface expression of IL-2Rα on blasts was reported to be an independent predictor of poor prognosis in AML [39], and IL-2Rα was also associated with chemotherapy resistance [40]. These results suggest that *WNT11*, *CCL2*, and *IL-2R*α upregulation in MDS blasts could be associated with disease progression by decreasing blast cell apoptosis.

TGF-β is a critical cytokine in the pathology of MDS because it was reported that overactivation of TGF-β signaling led to ineffective hematopoiesis [13] and inhibition of TGF-β signaling promoted hematopoiesis in MDS [12]. In addition to reports that plasma TGF-β1 levels were elevated in MDS patients [12, 41, 42], TGF-β1 has been implicated in the induction of other myelosuppressive cytokines such as IL-6, TNF, and IFN-γ, which may be involved in MDS pathogenesis and disease progression [43]. TGF-β1 may induce Tim-3 on MDS blasts (Figure 2D-2H) and could be involved in tumor proliferation as well as ineffective hematopoiesis.

In conclusion, these data suggest that Tim-3 expression on MDS blasts may be associated with disease progression of MDS characterized by tumor proliferation. These findings provide new insight into potential immunotherapy targeting the galectin-9–Tim-3 pathway.

## MATERIALS AND METHODS

### Patients and cell lines

BM and PB samples were obtained from patients with MDS and AL-MDS. The patients were diagnosed according to French-American-British (FAB) and WHO classifications. In addition, all patients were classified according to the IPSS and WPSS. Plasma samples were obtained by centrifugation of heparinized PB and stored at –20°C until use. Mononuclear cells (MNCs) were separated from BM and PB samples with Histopaque (Sigma-Aldrich, St. Louis, MO) density centrifugation. Written informed consent for study participation was obtained from each patient, and the study protocol was approved by the Institutional Review Board of Nippon Medical School. The human MDS cell lines F-36P (Riken Cell Bank, Ibaraki, Japan), SKM-1 (Health Science Research Resources Bank, Osaka, Japan), and MDS-L (kindly provided by Dr. Tohyama, Kawasaki Medical School, Okayama, Japan) [44] were cultured in complete medium consisting of RPMI 1640 medium (Wako Chemical Industries, Osaka, Japan) containing 10% fetal bovine serum, 100 U/ml of penicillin, and 100 mg/ml of streptomycin (Life Technologies, Carlsbad, CA) at 37°C under 5% CO_2_. F-36P cells were cultured in complete medium supplemented with IL-3 (Wako Chemical Industries) 5 ng/ml and MDS-L cells with IL-3 20 ng/ml. The human BM stromal cell line HS-5 was obtained from the American Type Culture Collection (Manassas, VA). The cell culture supernatant was harvested after 4-day cultivation with HS-5 cells and stored at –20°C until use.

### Reagents

STAT3 inhibitor V and pyrrolidine dithiocarbamate (NF-κB inhibitor) were purchased from Merck Millipore (Darmstadt, Germany). LY294002 (PI3K/Akt pathway inhibitor), AG 490 (JAK2 inhibitor), U0126 (MEK inhibitor), and SD208 (TGF-β receptor type 1 kinase inhibitor) were from Wako Chemical Industries. The concentration of inhibitors was determined when inhibitors had the greatest effect and least cytotoxicity, as previously reported [45]. IFN-γ, TNF-α, GM-CSF, MIP1-α, IL-10, IL-1β, and VEGF were also from Wako Chemical Industries.

### Enzyme-linked immunosorbent assay

Galectin-9 and HMGB-1 concentrations were measured using a Quantikine ELISA kit (R&D Systems, Minneapolis, MN) and HMGB-1 ELISA Kit II (Shino-test, Kanagawa, Japan), respectively, according to the manufacturers’ instructions. The lower limit of detection limit of galectin-9 and HMGB-1 using these kits is 0.008 and 1 ng/mL, respectively.

### Flow cytometry and cell sorting

Immunophenotyping was performed as described previously [3]. In brief, after blocking with human gamma globulin (Ig) (MP Biomedicals, Solon, OH), cells were stained with phycoerythrin (PE)-conjugated antibody for CD34, peridinin chlorophyll protein (PerCP)-conjugated antibody for CD45, and allophycocyanin (APC)-conjugated antibody for Tim-3 (BioLegend, San Diego, CA). Data acquisition was conducted in a FACSVerse flow cytometer (BD Biosciences, San Jose, CA) using Flowjo software (Tree Star, Inc., Ashland, OR). Isotype-matched negative control FITC- and PE-conjugated antibodies were used. MDS blasts were gated using the CD45-gating method, and the gated cells were confirmed by CD34/CD45 gating. At least 100,000 cell events were acquired for most samples [46]. Tim-3 expression was examined on the gated blasts. The relative mean fluorescence intensity (MFI) expression of Tim-3 was the ratio between the MFI of anti-Tim-3 antibody staining and the MFI of control IgG staining, as previously reported [47]. In cell sorting, after staining APC-conjugated anti-Tim-3 antibody, Tim-3^+^ and Tim-3^–^ F-36P cells were purified with an FACS Aria II cell sorter (BD Biosciences).

### Gene expression assay

Total cell RNA was extracted from cultured cells and PBMCs using TRIzol Reagent (Life Technologies), and cDNA was synthesized using Prime Script II Reverse Transcriptase (Takara Bio Inc., Shiga, Japan). The mRNA expression of *Tim-3* and *galectin-9* was determined in the quantitative real-time polymerase chain reaction (qPCR) using SYBR Premix Ex Taq II (Tli RNaseH Plus; Takara Bio Inc.) and 7500 Fast Real-Time PCR system (Life Technologies). The relative expression was calculated by the formula 2^-ΔCt^ x 1000. The ΔCt value was calculated by the subtraction of Ct values (target gene – internal control gene); β-actin was used as an internal control. Primer sequences are shown in Supplementary Table 1. The differential gene expression between Tim-3^+^ and Tim-3^–^ F-36P cells was identified using a SurePrint G3 Human Gene Expression 8x60K v3 microarray, which is comprised of probes corresponding to 26,083 gene RNAs and 30,606 long noncoding RNAs (Agilent Technologies, Santa Clara, CA).

### Intracellular Ki-67 staining

F-36P cells (2.0 × 10^5^) were co-cultured with 20 μg/ml of Ultra LEAF-purified anti-Tim-3 antibody (Biolegend) or isotype control for 30 min at 4°C. After overnight incubation, intracellular Ki-67 expression levels were measured with the FITC Ki-67 Mouse Anti-Ki-67 Set (BD Biosciences, San Jose, CA) according to the manufacturer’s instructions.

### Western blotting analysis

Cells were lysed with 2x SDS loading buffer (100 mM Tris-HCl, pH 6.8, 4% SDS, 0.004% bromophenol blue solution, 20% glycerol). Cell lysates were electrophoresed on SDS 12.5% polyacrylamide precast gel and transferred to a PVDF membrane. The ECL Prime Western Blotting Detection Kit (GE Healthcare Japan, Tokyo, Japan) was utilized to detect target proteins. The first antibodies were 1000-fold diluted rabbit anti-Tim-3 and 3000-fold diluted rabbit anti-β-actin antibody (Cell Signaling Technology Inc., Danvers, MA). Target protein chemiluminescence was detected using an imageQuant LAS 400 mini (GE Healthcare Japan) with ImageJ version 1.50i software (National Institutes of Health, Bethesda, MD).

### Statistical analysis

The Student *t* test, Mann-Whitney U test, and χ^2^ test were used to analyze the two-group comparisons. OS time was measured from the date of diagnosis to the date of the last follow-up or death as a result of any cause and estimated using the Kaplan-Meier method. The difference between the curves according to galectin-9 levels was assessed using the log-rank test. The Cox proportional hazard model was used to determine hazard ratios. *P* values of less than 0.05 were considered to represent a statistically significant difference.

## SUPPLEMENTARY MATERIALS FIGURES AND TABLES


