# Supplementary material for: Functional expression of Tim-3 on blasts and clinical impact of its ligand galectin-9 in myelodysplastic syndromes
Source: Oncotarget. 2017 Oct 4;8(51):88904–17. doi: 10.18632/oncotarget.21492 (PMC5687656; doi:10.18632/oncotarget.21492)
Supplement: Supplementary file 1 [file oncotarget-08-88904-s001.pdf]

Functional expression of Tim-3 on blasts and clinical impact of its ligand galectin-9 in myelodysplastic syndromes

SUPPLEMENTARY MATERIALS

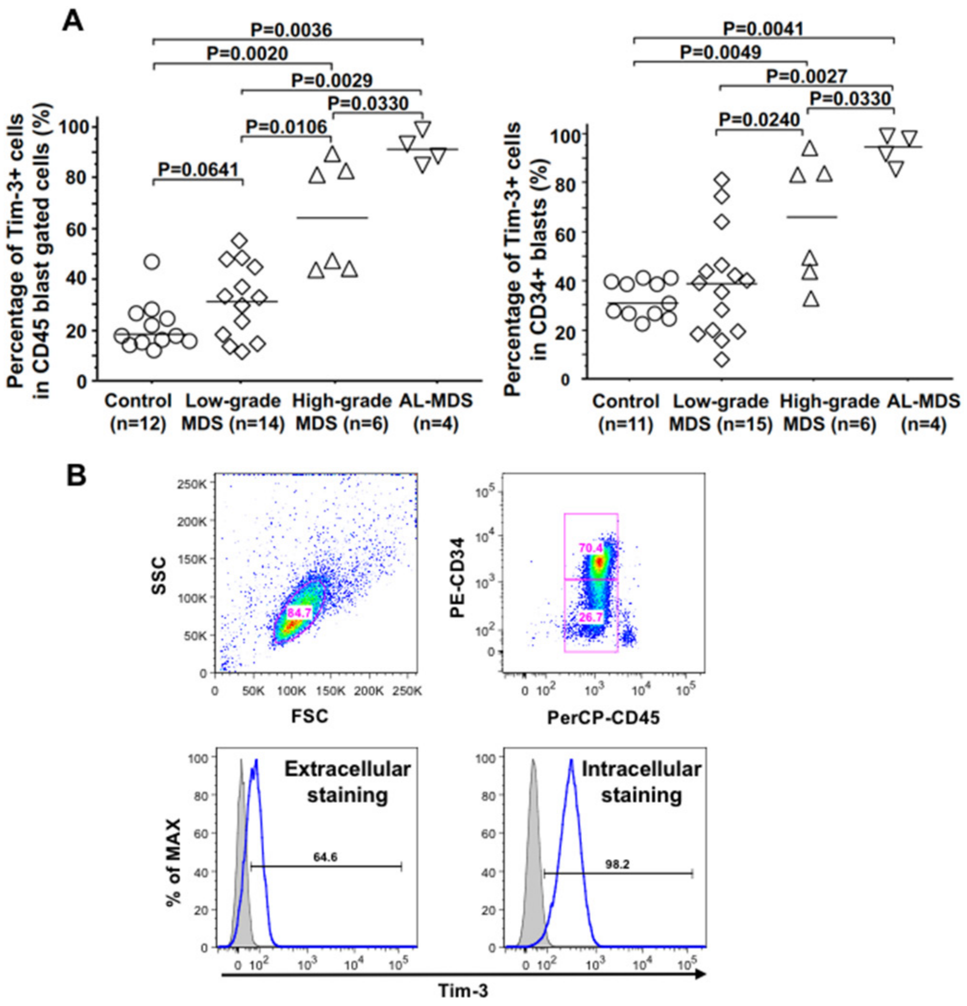

Supplementary Figure 1: Tim-3 expression on blasts obtained from patients with MDS and MDS/AL. (A) Comparison of cell surface Tim-3 expression on blasts, which were gated by CD45/side-scatter (left) and CD34/CD45 (right) gating, with those from normal controls and patients with MDS and AL-MDS. **(B)** Extracellular and intracellular Tim-3 expression levels In CD34/CD45 gated blasts from an AL-MDS patient.

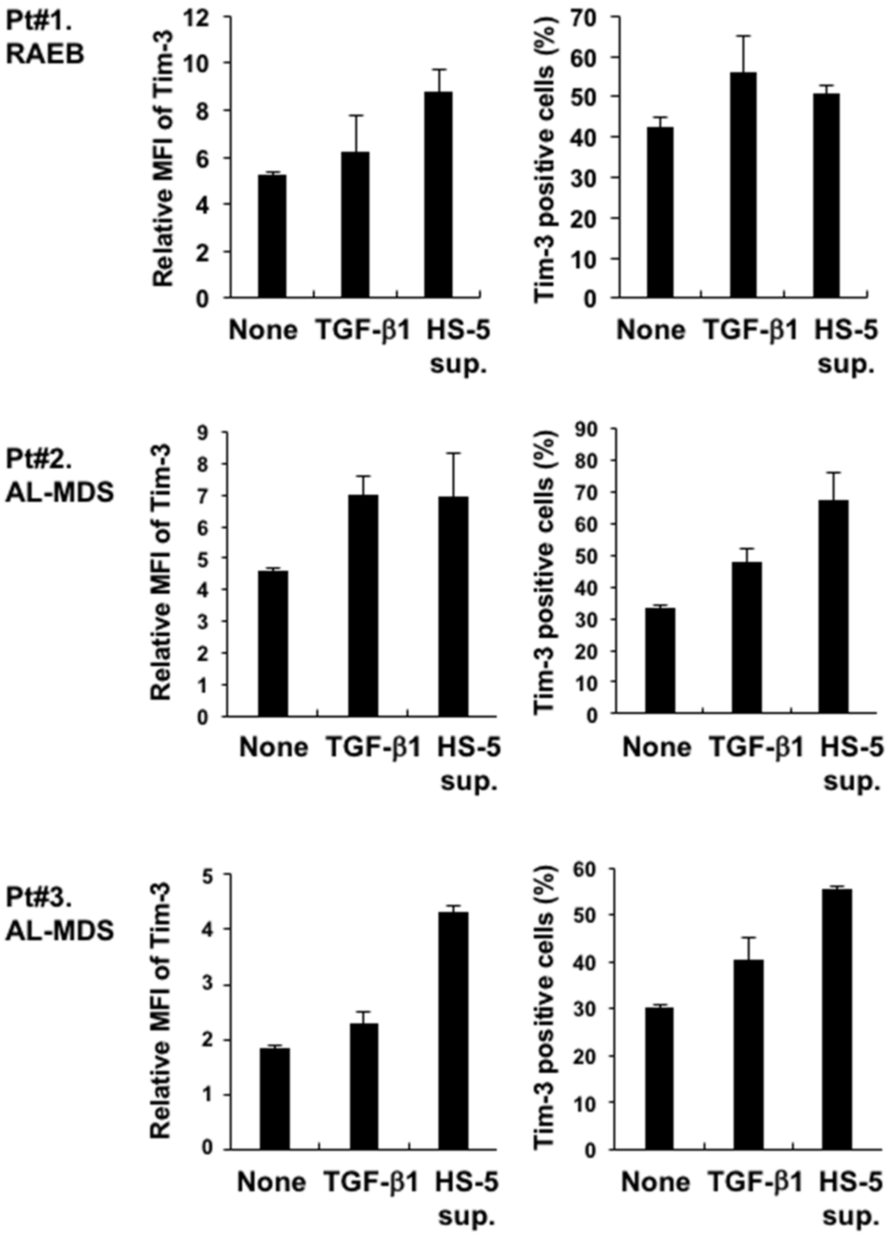

Supplementary Figure 2: Tim-3 induction on MDS blasts from patients. Bone marrow mononuclear cells obtained from 3 patients with RAEB and AL-MDS were cultured in the presence of TGF-b or HS-5 sup. for 2 days, and then Tim-3 expression levels on MDS blasts were analyzed by FCM.

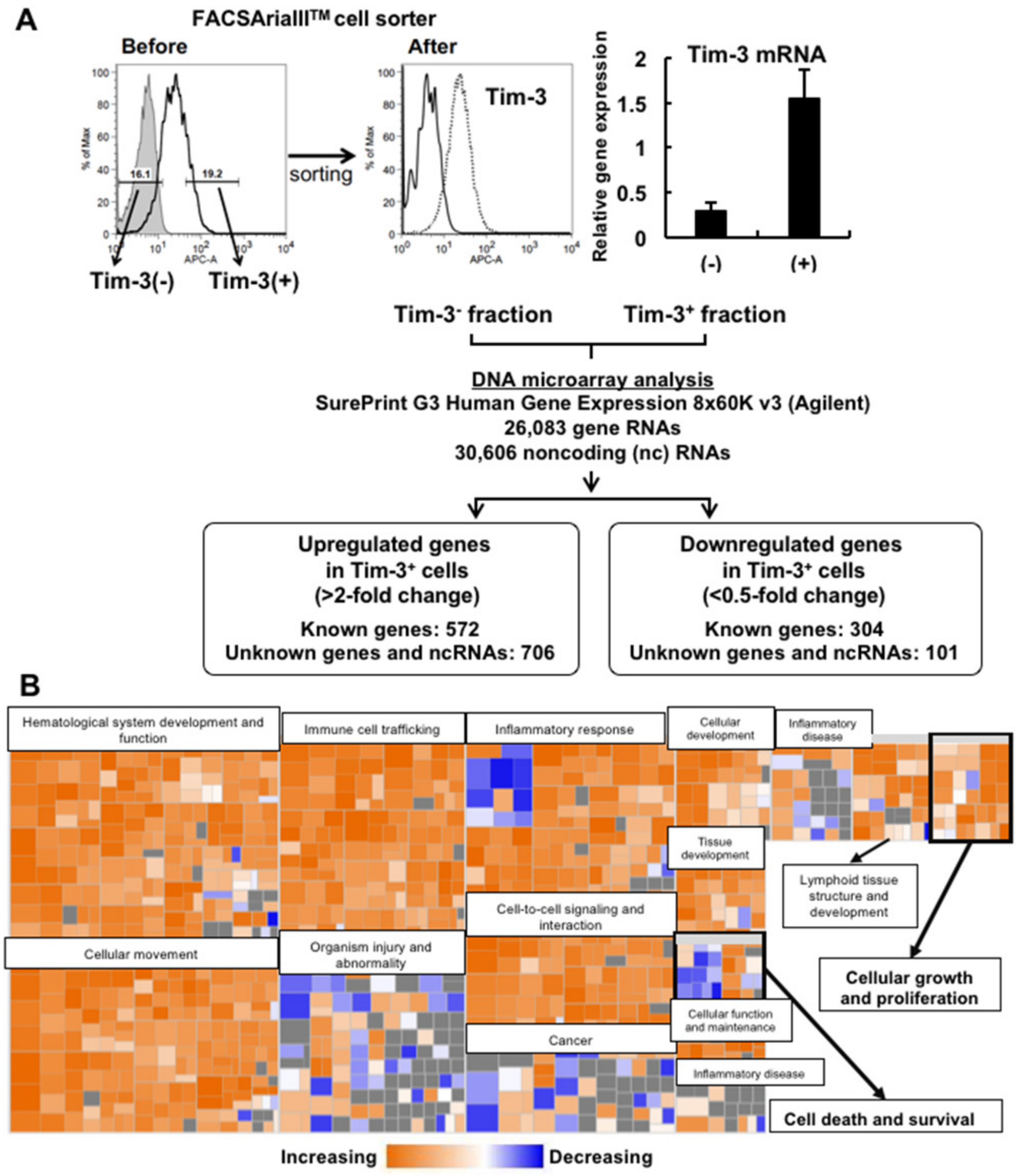

Supplementary Figure 3: Gene expression of Tim-3⁺ MDS blasts. After sorting, surface Tim-3 expression and Tim-3 mRNA were analyzed in the Tim-3⁺ fraction (histogram; dotted line) and Tim-3⁻ fraction (solid line) by FCM and real-time PCR, respectively **(A)**. Then DNA microarray analysis was performed **(B)**.

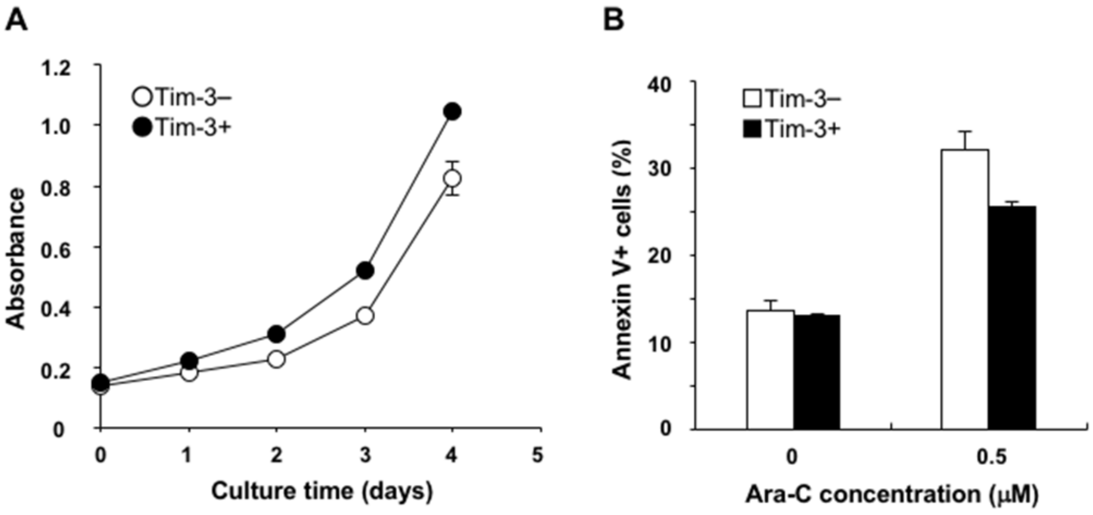

Supplementary Figure 4: Proliferative potential and drug resistance of Tim-3$^+$ MDS cells. Tim-3$^+$ and Tim-3$^-$ F-36P cells were isolated with an FACSAriaIII cell sorter and cultured in the presence of galectin-9. **(A)** Cell proliferation was determined in the WST-8 assay using the Cell Counting Kit-8 (CCK-8; Dojindo, Kumamoto, Japan) on days 0–4 of culture. **(B)** Annexin V$^+$ apoptotic cells in cell fractions induced by cytarabine (Ara-C) were determined by FCM.

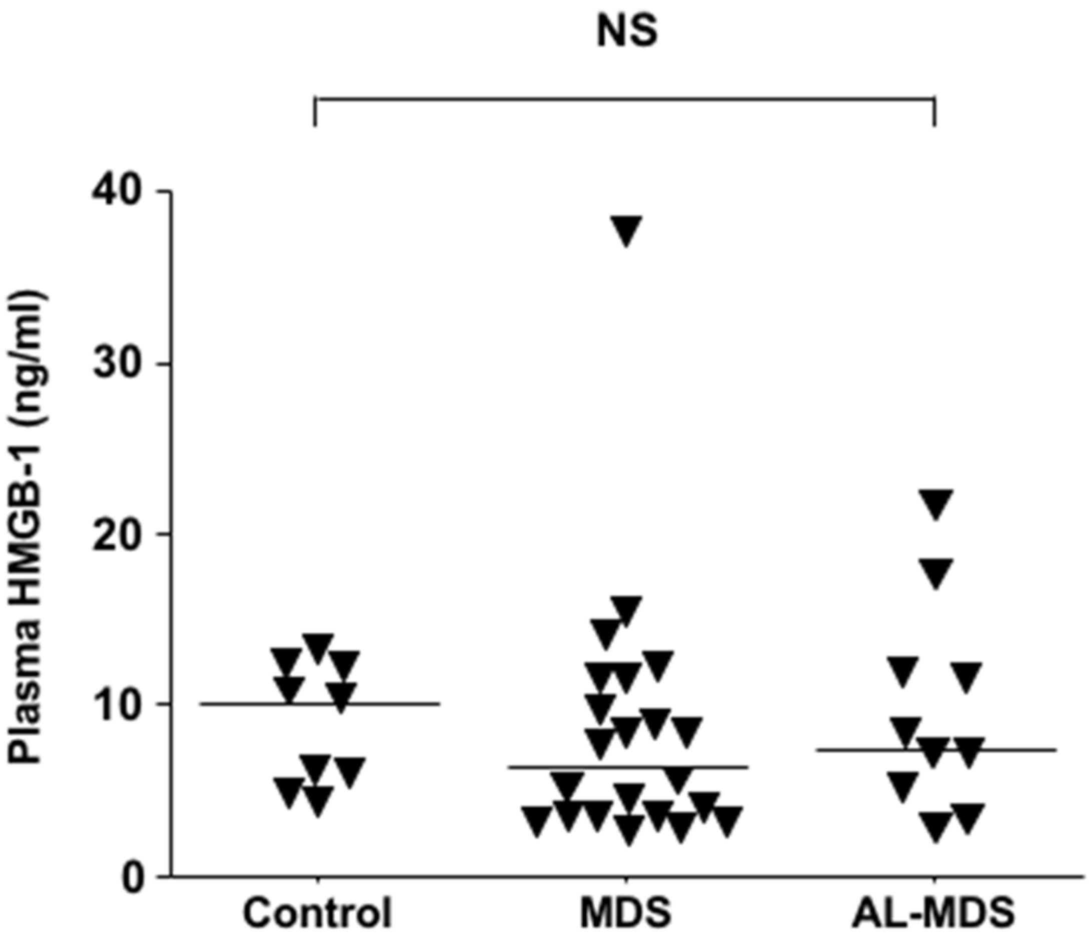

Supplementary Figure 5: Plasma HMGB-1 levels in patients with MDS/AL-MDS. HMGB-1 concentrations in plasma from 9 healthy controls, 22 MDS patients, and 10 AL-MDS patients.

Supplementary Table 1: Primer sequences

Accession no.	Gene symbol	Gene title	Primer sequence (5'-3') forward (upper) reverse (lower)
NM_001101.3	ACTB (β-actin)	Actin beta	TGGCACCCAGCACAATGAA<br>CTAAGTCATAGTCCGCCTAGAAGCA
NM_032782.4	HAVCR2 (Tim-3)	Human hepatitis A virus cellular receptor 2	ATTTCCGCAAAGGAGATGTG<br>ACCTTGGCTGGTTTGATGAC
NM_002308.3	LGALS-9 (galectin-9)	Galectin 9	AAGATGGAGGGTACGTGGTG<br>ATCCCGTTCACCATCACCTT
NM_001008540	CXCR4	C-X-C motif chemokine receptor 4	CTGGCCTTCATCAGTCTGGA<br>TCATCTGCCTCACTGACGTT
NM_001206866	IL6R	Interleukin-6 receptor	GTGCCAGTATTCCCAGGAGT<br>CAGGCTGCAAGATTCCACAA
NM_000584	CXCL8	C-X-C motif chemokine ligand 8	CAGTTTTGCCAAGGAGTGCT<br>CCAGTTTTCCTTGGGGTCCA
NM_002982	CCL2	C-C motif chemokine ligand 2	GCAGCAAGTGTCCCAAAGAA<br>TCGGAGTTTGGGTTTGCTTG
NM_004626	WNT11	Wnt family member 11	AGCTCAACCTGACTTCTGCAT<br>CACATAAGGTCGCAGCTGTC
NM_000417	IL2RA	Interleukin-2 receptor subunit alpha	CAAAGTCCAATGCAGCCAGT<br>ATAAACCATCTGCCCCACCA
